# Navigating rare disorder healthcare in Aotearoa New Zealand: an interpretative phenomenological analysis

**DOI:** 10.3389/frhs.2026.1822565

**Published:** 2026-06-30

**Authors:** Lucy Bennett, Tara N. Officer

**Affiliations:** Te Puna Hauora – School of Health, Te Herenga Waka – Victoria University of Wellington, Wellington, New Zealand

**Keywords:** Aotearoa New Zealand, health care navigation, health services research, interpretative phenomenological analysis, rare diseases, rare disorders, patient experience, relationship-centred care

## Abstract

**Introduction:**

Rare disorders collectively affect around 300 million people worldwide. They vary in aetiology, symptomology and treatment, yet people living with them experience many of the same diagnosis, information access, healthcare access, and support challenges. This research explores the lived experiences of navigating networks of care for people with rare disorders and their carers. Prior research in this area, particularly in Aotearoa New Zealand, is scant, meaning that it is difficult to clearly understand experiences of navigating rare disorder care and how they might be improved.

**Methods:**

Relational mapping interviews were undertaken with 11 people with rare disorders and four carers, recruited through the Rare Disorders New Zealand Facebook page. Participants described their networks of care and drew maps to visually represent them. The analysis of this data was informed by Interpretative Phenomenological Analysis.

**Results:**

Participants described the support available to them in the healthcare system through four main themes: the Support Empty Space, caring care, begging and paying for care, and feeling left behind by the system. These themes highlight challenges navigating fragmented healthcare systems, leading to a disconnect between participants' experiences of healthcare systems and their engagement with individual healthcare professionals.

**Discussion:**

Our findings suggest a need for greater system accountability for rare disorders healthcare navigation, reinforcing existing rare disorders research insights. Encouraging relationship-centred principles in rare disorder healthcare delivery and designating a place within health systems for rare disorder management and support are two steps that may support improved navigation experiences. Such steps may also reduce reliance on luck as a mechanism to engage with knowledgeable clinicians and help ensure necessary access to support.

## 1 Introduction

Living with a rare disorder is not a rare experience. There are over 10,000 different rare disorders; they include, for example, genetically inherited, neurological, metabolic, autoimmune, and developmental conditions, many of which are chronic, multisystemic, and associated with disability. Often defined as being a disorder affecting less than or equal to one in 2,000 people (1), collectively rare disorders affect around 300 million people worldwide (6% of the world's population) (2). Half of all rare disorders affect children; this population has a 30% mortality rate before five years of age (2). In Aotearoa New Zealand (Aotearoa), it is estimated that rare disorders affect around 300,000 people (3). Despite differences in the aetiology, symptoms and treatments of each disorder, people affected by them often face similar challenges, such as delayed diagnoses, poor information access, and challenges in accessing appropriate healthcare and support (4). Such delays in diagnosis further lead to under-reporting of the true prevalence of rare disorders, a problem contributing to challenges in defining rare disorders (5).

People living with rare disorders (PLWRD) engage with healthcare services more frequently than the general population, yet face barriers including geographical distance, financial strain, limited service availability, referral inefficiencies, and long wait times (6–8). Rare disorder care typically requires the involvement of multiple medical specialists and teams, yet service coordination remains inadequate, often forcing PLWRD and carers to manage their own care pathways (9). The absence of formal coordination results in numerous, often redundant appointments that fail to progress care (10, 11). Patients see one specialist at a time, repeating assessments that have already been conducted, further delaying healthcare outcomes (12–14). In contrast to more prevalent long-term conditions with established care pathways, therefore, PLWRD may rely more heavily on self-advocacy, informal networks, and navigation across multiple disconnected systems (15).

Alongside these logistical challenges, PLWRD and carers report dissatisfaction with healthcare professionals (HCP), who they describe as often showing arrogance, condescension and dismissiveness (16–19). Dissatisfaction with HCP is commonly linked to their failure to listen (18, 20), insufficient rare disorder knowledge (16, 19, 21), and reluctance to collaborate with other providers (10, 22).

Research about the care experiences of PLWRD and carers specific to Aotearoa is sparse, though a 2023 survey offers some insights into these experiences (23). PLWRD in Aotearoa demonstrate high healthcare engagement, with 85% consulting a specialist and 91% visiting a GP within the 70 days prior to completing the survey. Despite this, many perceive support services as inadequate: 51% reported accessing services that were poorly prepared to help, 64% described limited knowledge about their disorders within services, and over 70% felt that they lacked awareness of their rights, social services, and financial entitlements. Financial strain was also prevalent, with 60% reporting high disorder-related expenses and 54% struggling to manage them, while 74% experienced reduced income. Additionally, 36% reported often experiencing unhappiness or depression – compared to 11% in the general population – and 34% often felt unable to overcome their problems.

The *Aotearoa New Zealand Rare Disorders Strategy*, published by the Ministry of Health in 2024, sets a direction for the healthcare system to enhance support for PLWRD and their family (24). The strategy underscores the importance of integrating lived experiences into decision-making as the strategy is implemented. It also aligns with broader global recognition of rare disorders, such as the 2021 UN Resolution on Persons Living with a Rare Disease (25), and the 2025 World Health Assembly resolution ‘Rare diseases: a global health priority for equity and inclusion’ (26).

While challenges in healthcare access and delivery are recognised in international literature, there is very little evidence about how they are experienced in Aotearoa. This research aims to contribute to an increasing awareness of rare disorders by examining the lived experiences of PLWRD and carers in Aotearoa, guided by the following research questions:

1. What are the lived experiences of navigating networks of care for people living with rare disorders and their carers in Aotearoa?
2. How do people living with rare disorders in Aotearoa and their carers perceive strengths, weaknesses, and opportunities for change within these networks?

## 2 Materials and methods

This research employs Interpretative Phenomenological Analysis (IPA) as its methodology. Rooted in phenomenological, hermeneutic, and idiographic theories, IPA facilitates in-depth exploration of complex topics (27). Phenomenology emphasises the lived experiences of individuals (27–29), making it particularly relevant for understanding the perspectives of PLWRD and carers. Hermeneutics enables an examination of how these experiences are interpreted, allowing researchers to explore their depth and complexity (30, 31). This is especially valuable when studying populations that are often overlooked and underserved (30, 31). The idiographic focus of IPA further ensures an in-depth examination of a specific group's experiences rather than broad generalisations (27, 31). Overall, IPA researchers investigate how individuals make sense of their lived experiences (32).

IPA is well-suited for research on complex and emotionally charged topics (30), such as navigating rare disorder care. Previous IPA studies involving PLWRD have highlighted the intricate psychological, social, and contextual aspects of these experiences (e.g., 30–32). These insights are instrumental in informing caregivers, HCP, and policy developers on how to provide more meaningful and effective support to PLWRD and carers (33–35).

### 2.1 Recruitment

Following receipt of ethics approval (#2023/HE030868) from the Victoria University of Wellington Human Ethics Committee in May 2023, participants were recruited via an invitation posted on the Facebook page of Rare Disorders New Zealand (RDNZ), the national representative body for PLWRD and their families. Prospective participants then contacted the primary author (LB) directly via email or through a Microsoft Forms form to arrange a time for an interview. Eligibility was open to adults (18 years and older) residing in Aotearoa who either lived with or cared for someone with a formally diagnosed rare disorder. The inclusion of PLWRD and carers reflects the project's focus on navigating rare disorder care rather than the lived experience of rare disorders alone. Given that approximately 50% of rare disorders affect children (2), carers play a crucial role in managing healthcare access and coordination within the rare disorder community.

### 2.2 Data collection

Forty-five people indicated an interest in participating, of these, fifteen participants were interviewed for the project; these interviews took place between June and July of 2023. Potential participants were initially retained in a recruitment pool while the depth and diversity of experiential accounts was determined. The decision to include fifteen participants was pragmatic but also reflected a sample bigger than in typical IPA research (27).

All participants provided written informed consent prior to participating. LB conducted all interviews; these lasted between 33 and 80 min. Nine were Zoom (Zoom Communications Inc.) based; three were in person and three were over the phone, reflecting participants’ preferences. A field log was kept throughout data collection and analysis to support the ethical, methodological, and reflexive practices used.

Participants selected their own identifier and provided demographic information (Table 1). Participants were not asked to disclose their rare disorder, though many did during the course of the interview. Due to the Aotearoa population being small (just over 5 million), disorders have not been specified within this research. As only a few people may have each individual rare disorder, disclosing them would make participants potentially identifiable.

**Table 1:** Participant overview.

Identifier	Age range (years)	Ethnicity	Gender
Person living with a rare disorder			
Alison	45–54	British	Female
Chris	55–64	Australian	Male
Gill	55–64	New Zealand European	Female
Hilary	65+	New Zealand European	Female
J	55–64	Not disclosed	Female
M Weasel	25–34	New Zealand European	Female
Mrs Bucket	45–54	New Zealand European	Female
Rachel	35–44	New Zealand European	Female
Rose	55–64	New Zealand European	Female
Stella	35–44	Latin American	Female
Steve	45–54	New Zealand European	Male
Carer			
Anna	45–54	New Zealand European	Female
Dylan	35–44	New Zealand European, German	Female
John	65+	New Zealand European	Male
Rog	55–64	New Zealand European	Male

All participants had engaged with Aotearoa's healthcare system for the management of their rare disorder. Among the PLWRD, J, M Weasel, Mrs Bucket, Gill, Chris, Rose, and Stella had lived with their condition for more than a decade. Rachel had lived with her rare disorder for 6–10 years, while Steve and Alison had been diagnosed within the past five years. The four participants who were carers provided care for their own children, whose ages ranged from early childhood to adulthood. Additionally, Steve was accompanied by a support person, Shelley, during his interview, who consented to her input being included.

Data collection was conducted using relational mapping interviews (36), a method that integrates semi-structured interviews with a drawing task in which participants map their healthcare networks. The mapping approach involved four structured touchpoints (36): participants first positioning themselves within their healthcare network (mapping the self), then drawing maps representing the people, services, and relationships involved in their care (i.e., mapping significant others in their healthcare network), then standing back to reflect on broader patterns, and finally, considering change over time/ to their map. Alongside this mapping, an iterative draw-talk-draw-talk format facilitated visualisation and discussion, enabling participants to articulate the complexities of their healthcare relationships in greater depth (37).

Paper and drawing materials were provided for in-person interviews, while for online interviews, participants chose between creating maps beforehand or using the Zoom Whiteboard feature during the interview. Participants who were interviewed over the phone created their maps and sent them to the interviewer prior to the interview. Interview materials for in-person interviews were chosen to align with Zoom Whiteboard options, ensuring consistency. Instructions were provided to prepare participants and alleviate potential concerns, following the recommendations of Boden & Larkin (36). All methods were pilot tested prior to the interview.

### 2.3 Analysis

This research gathered textual data (interview transcripts) and visual data (relational maps), which were analysed using IPA methods. Interviews were transcribed using Otter AI software (Otter.ai, version 2.18.3), deidentified and checked for accuracy by the primary author (involving listening to audio files against the first-pass transcript and then amending transcripts as appropriate), and reviewed to identify key insights. Although research indicates that the use of artificial intelligence supported transcription software can face challenges when working with people with speech differences (38), this did not impact our transcription process. Participants were also provided with the opportunity to review their transcripts, two participants contacted the primary author after the research conclusion to provide additional information to their transcript. While IPA does not follow a singular prescribed approach, it is guided by flexible analytical frameworks (27) shaping the development of the analytical process in this study.

Preliminary lists of Personal Experiential Themes (PETs) were created for each transcript, which were then consolidated into Group Experiential Themes (GETs). These GETs were coded within NVivo (Lumivero, version 12.1.1) and applied to the interview data, with notes capturing evolving interpretations. The most relevant GETs were identified, sometimes merged, and structured into thematic categories. To ensure the analysis remained grounded in participants’ experiences, earlier stages – particularly initial field notes – were revisited throughout and after the analytical process. Preliminary findings were refined iteratively through discussions within the research team, allowing insights from initial analyses to inform subsequent interviews.

In IPA, all data elicited through interviews are important (39) it was, therefore, important that relational maps were analysed separately (37), supporting triangulation. Questions drawn from a framework initially described in Boden & Eatough (40) and further developed in Boden et al. (37), were used to provide a structured approach to the analysis; questions included:

1. What are the overall characteristics of the map?
2. How is the participant represented?
3. How are other people represented?
4. How are relationships represented?
5. What is the overall tone and impression of the image?

Each map was analysed using a spreadsheet, incorporating 22 sub-questions addressing elements such as colour, symmetry, focus, symbolism, materials, and style. GETs were developed from the visual data and compared with GETs derived from the textual data. In turn, this analysis process supported an understanding of the complexity of systems that participants navigated (37). The interaction between these two forms of analysis informed the final results.

## 3 Results

The results discussed in this paper are drawn from participants’ mapping of important others in their interviews, and their reflections when they ’stood back’ from their relational maps. Four themes were identified that highlight how PLWRD and carers in Aotearoa experience healthcare: the Support Empty Space; caring care; begging and paying for care; and feeling left behind by the system.

### 3.1 The support empty space

A defining characteristic of participant maps was the overwhelming presence of empty space. Dylan described this as the “Support Empty Space,” referring to the areas where support was expected, but absent. This does not mean that the maps were sparsely populated – participants included anywhere from four to twenty groups or individuals – but rather, the connections between them were largely missing. Names were represented in isolation, scattered arbitrarily in blank space, reinforcing the absence of interconnected networks. Hilary, for instance, struggled to visualise a traditional map, choosing instead to construct a timeline, stating*,*

“I couldn't imagine a map, except as a long skinny line with things poking out… it's been, sort of over about five years, and not much interconnection between anyone.”

Her approach underscores the fragmented nature of support networks, in which individuals and resources exist, but fail to come together into a cohesive system.

**Figure 1:**
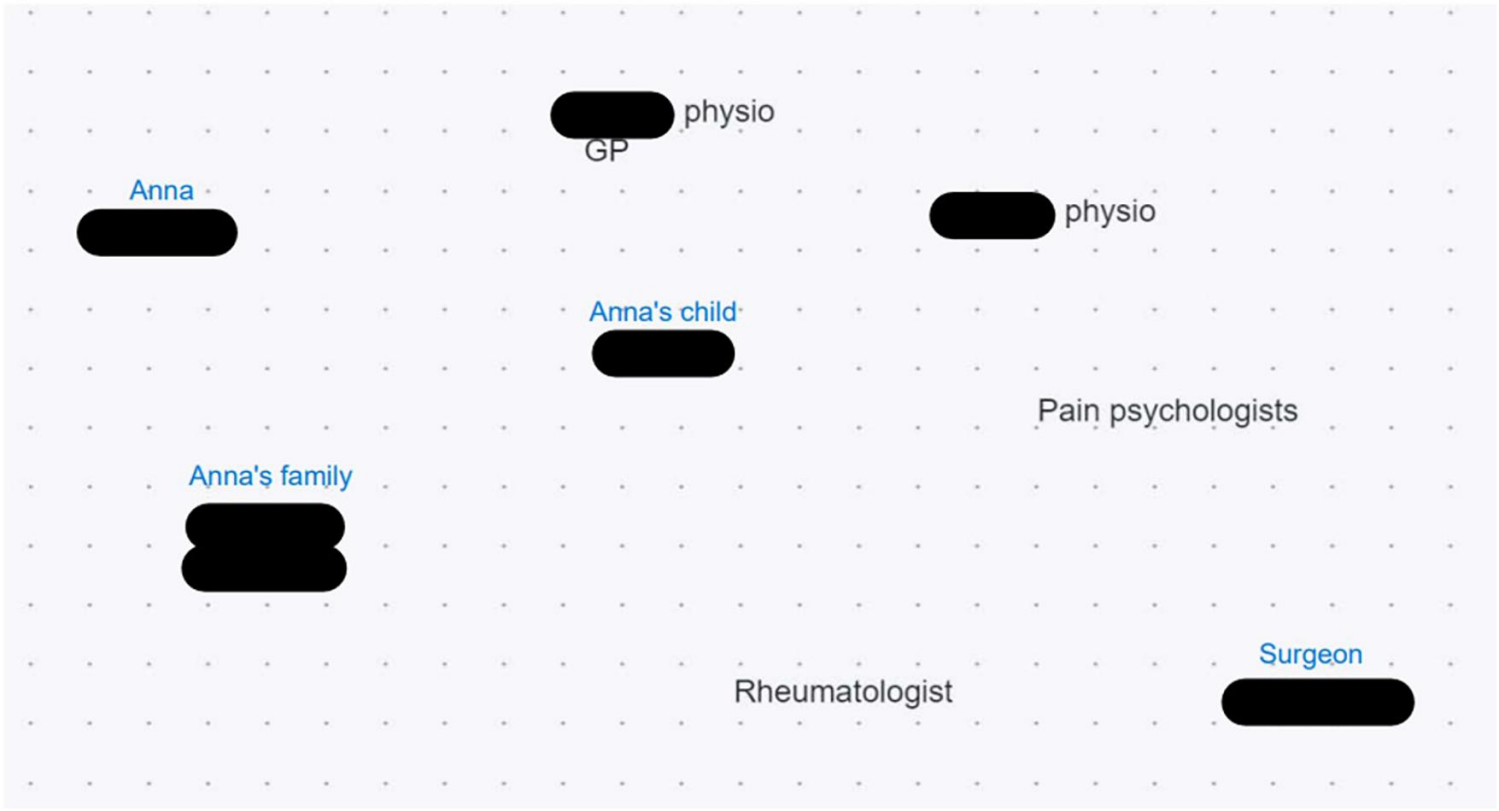
Anna’s relational map.

**Figure 2:**
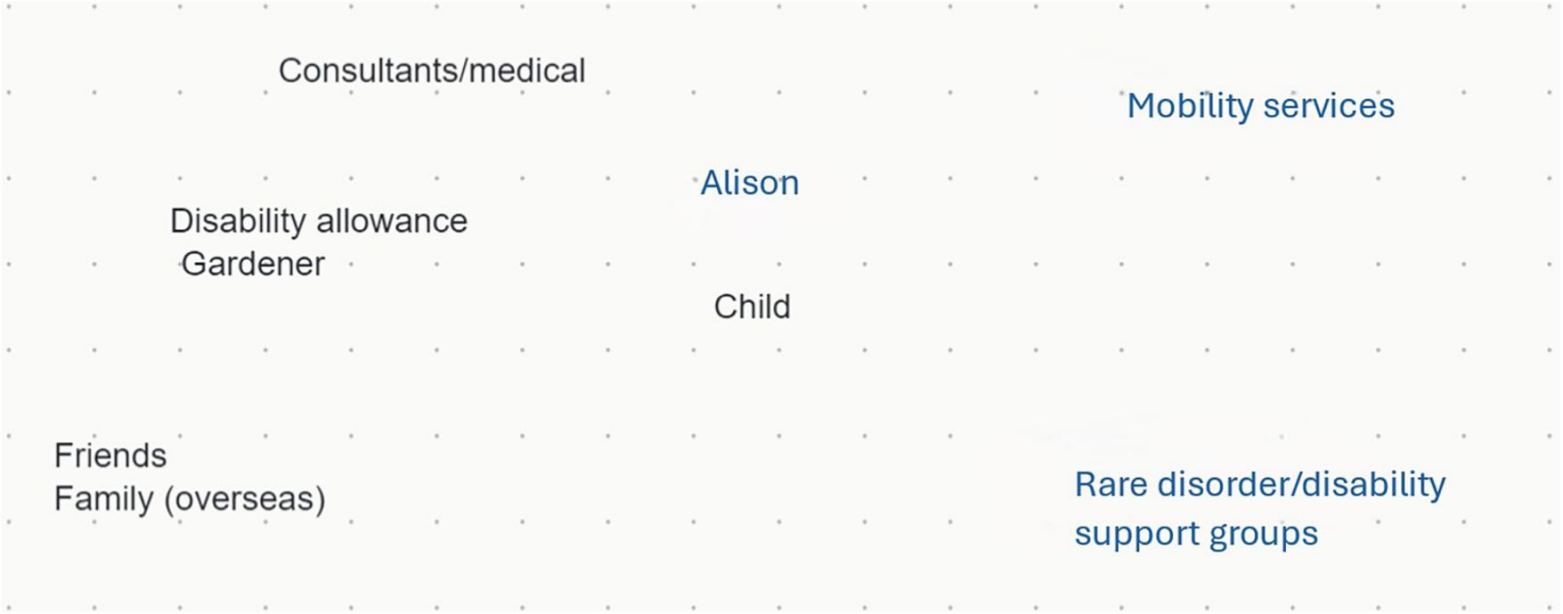
Alison’s relational map.

The Support Empty Space was evident in relational maps. Rather than being placed within a recognisable network, sources of support were isolated from one another, reinforcing a sense of disconnected care. Anna's map (Figure 1) and Alison's map (Figure 2) exemplify this. Despite including multiple different names and roles, these appear scattered across the Support Empty Space, without connections to each other or to PLWRD and carers. As a result, the maps themselves become dominated by empty space.

Participants’ descriptions of HCP illustrated their positioning within the Support Empty Space. Participants described interactions with multiple HCP who had varied roles, including medical treatment, psychological support, and pain management. However, the perceived significance of these professionals was determined less by their technical expertise and more by their attitude and manner. The distance between HCP and the participants on the maps often reflected the quality of their relationship – those positioned further away were more frequently described in negative terms, particularly concerning how they communicated information about rare disorders.

Anna's experience with a surgeon illustrates this disconnect. She relegated him to the lowest part of her map, recalling,

“He looked at [my daughter's] knee and said he wouldn't operate because there was nothing structurally wrong… he made the right call on that she shouldn't have an operation, but he told her to forget about the pain.”

While the decision itself may have been correct, the lack of empathy left Anna feeling dismissed and unsupported, reinforcing the feeling of empty space in which she was left to navigate next steps alone.

Dylan similarly described a conversation with a nurse, who stated matter-of-factly that the rare disorder was uncommon, implicitly justifying the lack of available support. Dylan expressed her frustration, pointing out,

“Just because it's not common doesn't mean that [my child] shouldn't be able to live like every other child.”

Despite recognising that the nurse was not intentionally insensitive, Dylan still felt abandoned in the Support Empty Space – a common feeling among participants interacting with HCP.

Shelley and Steve echoed these frustrations when discussing their experiences with specialists. While Steve eventually developed a good relationship with his primary specialist, his initial diagnosis was delivered bluntly, leaving no room for hope. Shelley described the emotional toll of being treated as just another case, recalling that, at the time, she wanted to shake the specialists and say,

“This is the most important thing happening in our lives, and we're just a 20-minute appointment to you.”

Over time, they found ways to carve out space for optimism, but the initial experience underscored how easily PLWRD can be left to fend for themselves within a system that does not prioritise their lived realities.

Ultimately, the Support Empty Space exists not only because of systemic constraints on HCP but also because of the way these professionals interact with PLWRD and carers. Even when HCP had the necessary medical expertise to provide care, participants described feeling uncared for due to a lack of supportive communication. This suggests that the disconnectedness of rare disorder care networks could be mitigated, to some extent, by HCP who prioritise empathetic communication and relational trust as core elements of clinical practice.

### 3.2 Caring care

Many participants described encounters with HCP who demonstrated care that felt caring, showing not just medical expertise in their interactions with PLWRD and carers, but also empathy, attentiveness, and respect. While negative experiences were part of their journey, they also recalled meaningful interactions where HCP took time to listen, engage, and genuinely support them.

John suggested that caring HCP are the norm, emphasising that while negative encounters tend to be more memorable, most professionals strive to be helpful. He noted,

“Overwhelmingly, health professionals were helpful, thoughtful, respectful, and gave it their best shot, you know? And I know that there's this tendency sometimes for people to feel that anger about the bad moments they have, but not remember that those are almost always a small minority.”

His perspective underscores that, even within systemic constraints, some HCP work hard to ensure patients feel heard and supported.

Other participants echoed this sentiment, recalling interactions that reassured them despite the challenges of living with a rare disorder. Anna, for example, described her child's physiotherapist as “*extremely helpful… he really listened and tried to do his best.”* This was a recurring theme – HCP were valued not for their ability to provide definitive solutions but for their willingness to engage, listen, and try. Participants did not expect cures or perfect guidance, but they deeply appreciated professionals who made an effort to understand their experiences.

Other participants similarly highlighted positive aspects of their healthcare professional relationships. Hilary praised her specialist for being “*very caring really, gentle,”* and appreciated the ability to contact him whenever she needed. J spoke warmly about her connection to the pharmacy, describing the pharmacist as “*really approachable, really helpful.”* Rose expressed appreciation for her GP, noting that “*he's never fought me once on anything… he's worked with me, not against me.”* She recognised that he did not always have answers but valued his openness and collaboration. Likewise, Stella described how her GP “*acts really fast and works really well with the [specialist],”* reinforcing the importance of responsiveness in effective care.

Rachel's experience was different, yet equally telling. While her specialist had a “*cold and clinical”* demeanour, she nonetheless felt gratitude for his medical expertise. After years of being dismissed, simply having someone validate her illness was profoundly meaningful:

“No one believed me for like 10 years… so just having someone sit there in front of me and say, yes, you have a disease. That feels like enough.”

Although he did not offer the empathy others sought, he provided the medical certainty she desperately needed. In her words, “*when you've been starving for so long, and someone gives you a crust, you're really grateful.”* Rachel's experience demonstrates that, for her, caring care is about recognition, validation, and the assurance her suffering is real. While she did not receive the warmth that other participants relied on, she was willing to sacrifice this for the “*crust”* of validation.

These experiences demonstrate that caring care does not require extensive resources or perfect solutions, but is evident in moments where HCP take time to acknowledge, listen, and support patients. However, these compassionate care instances often exist in isolation, within a fragmented healthcare system that struggles to foster consistent, interconnected support. While caring care improves the experiences of PLWRD and carers within healthcare interactions, it does not address systemic barriers that make access to support so precarious.

### 3.3 Begging and paying for care

Accessing care for rare disorders is rarely straightforward. Participants described a system in which pathways to support were fragmented, unreliable, and often dependent on personal advocacy, financial means, or sheer luck. When reflecting on the support available to her, Rachel observed,

“The underlying sentiment is that there isn't really much. Any support you do get, you have to beg and pay for. You have to beg and pay a lot of money for it.”

Despite appreciating the care they eventually received, participants observed that accessing that care overwhelmingly relied on precarious or unsustainable means.

One of the key ways participants accessed care was through private healthcare services. Rachel described her experience seeking help for her deteriorating vision:

“I went to the doctor, and I was like, something's wrong with my eyes and my brain. He referred me to neurology… I didn't hear from neurology for a full year, but I self-referred to an ophthalmologist because my health insurance covers it. I was on a medicine that could have made me blind. If I hadn't self-referred, and I heard from neurology, like a year later, I mean, I could have had visual damage.”

Her decision to bypass the public system may have prevented lasting visual damage, underscoring the risks inherent in waiting for public healthcare.

Others shared similar experiences. Steve and Shelley sought a private specialist after researching Steve's rare disorder and realising that, untreated, his life expectancy could be shorter than the time required to see a public specialist. Similarly, Dylan turned to the private system because the options she needed were unavailable in “*the normal system,”* and Alison noted that she had received “*nothing through the public system.”*

While private healthcare provided solutions, it also deepened financial strain. Anna tracked her daughter's medical costs in a spreadsheet, which had already totalled over $2,500 for the year. Rog estimated that without health insurance, they would be “*close to 100 grand out of pocket.”* While some participants had insurance, it did not remove financial anxiety. Rog, for example, had insurance to pay for surgical care, but was aware that “*you're then paying premiums that are through the roof, if you were to have every medical situation covered.”* Alison expressed concerns about the cost of magnetic resonance imaging, especially while unemployed, while Rachel admitted she was fortunate to afford physiotherapy, but still had to budget carefully for it. As Alison reflected, “*the support that mostly I have is because I've paid for it… the biggest support is because I've paid for them rather than that they're there for you.”* Even if the care that PLWRD receive is helpful, it often relies on PLWRD and carers having financial wherewithal.

Beyond financial barriers, participants also spoke of luck in accessing care. Anna credited a personal connection with securing an appointment for her daughter within days, where it could have otherwise been months before seeing someone. Rog described how his son's physiotherapist had a familial link to a specialist, and this personal relationship led to an accurate diagnosis, noting, “*the only reason we actually got the correct diagnosis was through pillow talk.”* The specialist at Steve's hospital happened to be a close friend of the leading expert for his rare disorder in the United States, which facilitated his diagnosis and care. Participants repeatedly described finding support through fortunate personal connections rather than structured healthcare pathways.

Shelley summed up the issue in her reflection that “*it's a bit scary how much luck came into it. You don't really want luck to be a main component of your health.”* No matter how skilled individual providers may be, if access to care depends on financial privilege or coincidence, then healthcare remains fragmented. These experiences illustrate the instability of existing pathways, requiring PLWRD and carers to navigate rare disorder care without reliable guidance.

### 3.4 Feeling left behind by the system

For many participants, the healthcare system felt like an abstract entity, detached from their actual care experiences. Anna described it as something “*out there,”* disconnected from the practicalities of treatment. J expressed a similar frustration, saying she felt “*totally left behind by the healthcare system,”* even though she appreciated the few providers who had supported her. She valued her GP, pharmacy, and specialist clinic, noting*, “somebody's there, somebody cares.”* Yet, her gratitude for these providers stemmed from a contrast – she felt abandoned by the system overall, which left her without continuity, follow-up, or practical support.

This isolation from the system was widespread. Stella acknowledged the responsiveness of her GP but framed it as exceptional rather than systemic, stating, “*I'm just really lucky that she's really responsive, that's just who she is as a doctor… I*’*ve heard that's not a normal response.”* Similarly, Anna described the way the system seemed to prioritise treatable conditions like cancer or heart disease while leaving those with rare, chronic illnesses to manage their own care: “*no one teaches you how to do that.”*

Some participants explicitly acknowledged the system's limitations rather than blaming individual HCP. Dylan, for instance, recognised the time constraints placed on professionals, stating, “*I don't necessarily blame them… but I think we just need to change our thinking.”* Rachel similarly noted that many problems stemmed from burnout and understaffing rather than intentional neglect. While they did not resent HCP, they believed that systemic reform was necessary to prevent patients from being left behind.

This depersonalisation of the system conveyed a sense of powerlessness, reinforcing the feeling of being left behind. The contrast between individual providers who demonstrated compassion, and the broader system that felt indifferent, contributed to the pervasive feeling that PLWRD and carers had to fight for recognition.

John and Shelley offered more forgiving perspectives, balancing the system's flaws with its advantages. John acknowledged genuine efforts to design good healthcare services, while Shelley described the healthcare system as “*relatively fit”* compared to other countries. However, both recognised that rare disorders fell through the cracks, reinforcing the need for a stronger, more inclusive framework.

Ultimately, participants felt that their relationship with the healthcare system was separate from their interactions with individual providers. While some HCP maintained connections and provided meaningful care, the system itself remained fragmented and impersonal. As Dylan summarised, “*as long as there's no issues, the system works well.”* But for PLWRD and carers, the system's shortcomings were deeply felt, leaving them struggling to access the care they needed. Their reflections highlight the need for structural changes to ensure that rare disorder care is not overlooked or left behind.

## 4 Discussion

Healthcare navigation experiences among PLWRD and their carers suggest that care access and service delivery is often achieved through individual perseverance rather than coordinated service delivery. Our study, involving relational mapping interviews with 15 participants, brought together four key themes: the Support Empty Space, caring care, begging and paying for care, and feeling left behind by the system. Many of these themes align with extant rare disorders literature. However, importantly, they provide contextual understanding of care in a publicly funded health system shaped by dispersed and diverse populations, workforce pressures, and ongoing restructuring (41), where the rarity of individual conditions may further fragment concentrated expertise and coordinated care pathways. Participants’ experiences reveal that access to care is shaped by luck and financial means, while quality of care is often determined by the attitudes of individual HCP. This demonstrates that systemic changes, rather than isolated improvements, are needed to ensure that PLWRD have access to reliable, consistent, and effective care. Research examining these experiences within Aotearoa remains limited.

The Support Empty Space emerged as a significant finding of this research, which was reflected visually through disconnected map structures, dispersed provider relationships, and participants positioning themselves as central coordinators of care within an empty space. Unlike networked care, which consists of interconnected individuals and services, support for rare disorders in Aotearoa is often detached, transient, and elusive. The Support Empty Space represents both the presence and absence of care, reflecting the fragmented and luck-driven nature of accessing healthcare services. This concept aligns with existing literature, which describes the uncoordinated nature of rare disorder care and the self-management burden placed on carers (9, 12, 42, 43). Moreover, the research revealed how PLWRD and carers conceptualise the Aotearoa healthcare system and their roles within it. They often felt supported by individual HCP, but perceived the system itself as lacking in support and leaving them behind. The research underscores the challenge of navigating fragmented, non-networked care systems, emphasising the need to establish visible places and support pathways specifically designed for individuals with rare disorders, and also highlighting the need for responsive wellbeing services that recognise PLWRD face challenges of invisibility, abandonment, and exclusion (13, 18).

However, this research identified certain strengths within the fragmented support system. Communication and empathy were highly valued by PLWRD and carers, even when HCP lacked disorder-specific knowledge. Positive perceptions of HCP arose when they showed compassion, attempted to help despite limitations, and believed what PLWRD and carers told them. This reinforces findings from Currie & Szabo (12), Fitzgerald et al. (44), and Gómez-Zúñiga et al. (45) regarding the importance of empathy and communication in rare disorder care. Participants’ experiences suggest that relational support from HCP can ease the emotional and practical burden of navigating disconnected rare disorder care.

A unique aspect of this research is the exploration of the means participants used to access healthcare, particularly the role of luck. Many described coincidental connections that led to timely support – sometimes through personal networks, chance encounters, or informal referrals. While this could reflect the benefits of living in a smaller country, it may also speak to participants’ desire to find something positive in an otherwise difficult experience. By framing access to healthcare as luck, rather than a failure where healthcare should have been provided as a right, some participants seemed to reclaim a sense of optimism, aligning with research around the impact of positive health outcomes amongst PLWRD on life perspectives (46). On the other hand, the sparse and inconsistently connected relational maps may also reflect the perceived precarity of relying on luck, where support was often dependent on isolated individuals rather than coordinated systems of care; findings also mirrored in other contexts (47). Ethically, this raises important questions around reasonable expectations of equitable access to healthcare services of an appropriate standard for PLWRD and their families.

Participants spoke about the need for personal advocacy and funding to receive timely and appropriate support. In several accounts, participants perceived accessing privately funded services as necessary to progress care, creating tension between healthcare need, ability to pay, and care continuity through participants accessing both public and privately funded health services. Although financial strain in rare disorder care and the impact on delayed health service access is well documented (11, 18, 22), the reality of having to pay for access, rather than receiving it as a right, as with much of Aotearoa's health services, is rarely explored. When care was not available through public systems, financial means replaced luck as a way to access timely support privately. Such a finding is particularly timely given increasing concerns around health service fragmentation in Aotearoa following the COVID-19 pandemic and ongoing health system reforms (48, 60). In turn, they suggest that the need to advocate for and financially support access to care are closely intertwined.

### 4.1 Implications for policy and practice

This research explores the healthcare navigation experiences of PLWRD and their carers in Aotearoa. Examining these experiences collectively highlights common challenges faced by this population, and focusing on the lived experiences of PLWRD and carers prioritises their voices to identify challenges and barriers accurately. Historically, PLWRD and carers have received limited attention from the healthcare system, and there are opportunities for improvement in Aotearoa's approach to caring for people with rare disorders (3). This research is a foundation for ongoing studies into their experiences, and underscores the need for policy and practice to better meet the needs of PLWRD and their carers.

Participants’ descriptions of caring care, wherein HCP took time to listen, engage, and empathise, reflect the core principles of relationship-centred care (RCC) (49, 50). Formally implementing RCC in rare disorder care may help transform caring care from an exception into a systemic norm, addressing the Support Empty Space identified by PLWRD and carers. By emphasising the importance of clinician-patient, clinician-clinician, and clinician-community relationships, RCC aligns with participants’ calls for empathy, continuity, and connection. In this way, RCC provides a structural pathway for embedding relational care into healthcare practice.

Formulating policies and structural support to enable the implementation of RCC at organisational levels could benefit PLWRD and carers. Measures could include resourcing healthcare organisations so that HCP can dedicate more time to patients, improving referral pathways and communication among HCP, and creating opportunities for HCP to interact with communities as part of their roles. Notably, such changes are hard to sustain within resource-constrained health systems facing workforce shortages, increasing service demand from an ageing population, and an increasingly fragmented service model. This risk, particularly concerning for PLWRD who already face coordination challenges, may place additional burdens on carers and those navigating the system if they are put into positions of navigational responsibility or if responsibility is left solely as a role for ‘caring’ HCP. At the least, RCC can also be integrated into professional education programs for both practicing HCP and students (50). Additionally, platforms that enable HCP to share knowledge and collaborate on rare disorder care could strengthen relational networks. For PLWRD and carers, interacting with HCP who practice relationship-centred care may significantly improve healthcare experiences.

To address the disconnection felt by PLWRD and carers from the overall healthcare system, findings highlight the importance of establishing clear pathways for navigating care. This aligns with broader rare disorder literature discussing coordinated service models, including the potential for centres of excellence to address fragmented care and dispersed clinical knowledge (51, 52). Such approaches have also been proposed nationally by RDNZ (53) and encouraged in the World Health Assembly resolution (26). RDNZ's proposal is based on an Australian model (54), and would be coordinated from a major city and include a virtual team of rare disorder experts across Aotearoa, extending their existing roles in healthcare. The centre would accept referrals from diagnostic services, offering multidisciplinary support and service coordination, alongside guidance for HCP. Additionally, the centre would engage in research and clinical trials, advocate for change, and highlight service provision gaps. As a recognised entity within the healthcare system, this centre could begin to fill the Support Empty Space by formalising connections between professionals and PLWRD, reducing the isolation participants described in their relational maps. The feasibility, however, of establishing such a centre in countries like Aotearoa, facing challenges of growing highly specialised clinical resources across diverse and individually rare conditions, raises broader questions about how healthcare systems prioritise care for populations whose needs may fall outside conventional service structures (55). Larger qualitative or mixed-methods health services studies examining care coordination, navigation, and relationship-centred support across diverse rare disorder populations in Aotearoa may strengthen the evidence base for centres of excellence and decisions around care delivery, informing future policy and service development.

### 4.2 Limitations and future research

International research points to limited exploration of compounding inequities in the rare disorders space (56). Indigenous Māori, and Pasifika or Asian populations often have worse healthcare access and service delivery in Aotearoa (57). Our research did not recruit participants from these populations. Further, our research predominantly involved female participants. To explore cultural and gender disparities in rare disorder care, recruiting a more diverse group of participants, and using Indigenous research methodologies should be focuses of future research.

This research focused on healthcare system experiences, but did not consider the specific processes and practices within healthcare systems shaping these experiences. Similarly, our research did not investigate the overlap between healthcare and other systems such as education and social welfare or disability support, despite this being an issue mentioned by participants. Future research would benefit from a focus on these aspects of rare disorder care, particularly when considering the life-long nature of many rare disorders.

As with all qualitative research, the purpose of this research is not generalisation. Acknowledging the small participant sample, particularly with relation to our carer sample, future research exploring the views of carers of PLWRD is timely. Having the views of this participant group, and the views of children living with rare disorders, is particularly important given the significant impact of rare disorders in paediatric populations (2).

Interview mode influenced participant engagement with relational mapping, with telephone participants completing maps independently, those providing physical maps engaging more with the process, and some appearing distracted by the mapping process. For PLWRD and concomitant disability, participatory visual data collection approaches, including relational mapping, may be particularly valuable given issues such as fatigue, communication preferences, accessibility needs, and the often complex, carer-inclusive nature of support. Such approaches may, therefore, facilitate inclusion of groups who would otherwise not participate in research (58). However, when considering undertaking such research, researchers are invited to consider the purpose of the task. For example, participants completing relational maps independently may have had greater opportunity for reflection, discussion with family, and pacing, whereas real-time mapping enabled immediate clarification and meaning co-construction. However, online formats may have also constrained the flexibility and diversity of relational map creation or led to challenges for population subsets compared with paper-based approaches (59), further complicating analysis processes. These findings highlight the need for further research on participatory visual approaches within healthcare-related research in rare disorder populations, particularly as an opportunity for assessing the value of relational mapping for capturing the perspectives of multiple people involved in care.

## 5 Conclusion

This research highlights the challenges facing PLWRD and their carers in Aotearoa. By exploring the lived experiences of navigating networks of care, the research has identified how PLWRD and carers experience Aotearoa's healthcare system, and where strengths and weaknesses lie. The findings underscore a need for systemic change to enhance the quality and coordination of care within the healthcare system, ensuring that PLWRD and their carers are not left behind. Implementing relationship-centred care and establishing clear holistic pathways for navigating rare disorder care, such as through a centre of excellence, are essential steps towards creating a more inclusive and supportive healthcare environment.

## Data Availability

The datasets presented in this article are not readily available to protect confidentiality, in line with the ethical consent provided by participants. Requests to access the datasets should be directed to the Chair of the Victoria University of Wellington Human Ethics Committee (human-ethics@vuw.ac.nz).
